# Secretome as neuropathology-targeted intervention of Parkinson’s disease

**DOI:** 10.1016/j.reth.2022.08.003

**Published:** 2022-08-28

**Authors:** Christian Ardianto, Robert Shen, Jimmy F.A. Barus, Poppy Kristina Sasmita, Yuda Turana, Lilis Lilis, Veronika Maria Sidharta

**Affiliations:** a Department of Histology, School of Medicine and Health Sciences, Atma Jaya Catholic University of Indonesia, Indonesia; b Master Program in Biomedical Sciences, School of Medicine and Health Sciences, Atma Jaya Catholic University of Indonesia, Indonesia; c Department of Neurology, School of Medicine and Health Sciences, Atma Jaya Catholic University of Indonesia, Indonesia; d Department of Anatomy, School of Medicine and Health Sciences, Atma Jaya Catholic University of Indonesia, Indonesia; e Department of Anatomical Pathology, School of Medicine and Health Sciences, Atma Jaya Catholic University of Indonesia, Indonesia

**Keywords:** Mesenchymal stem cell, Molecular targeted therapy, Neurodegenerative disease, Regenerative medicine, Synucleinopathy

## Abstract

Parkinson’s disease (PD) is the second most common progressive neurodegenerative disease, characterized by apoptosis of dopaminergic neurons in substansia nigra pars compacta (SNpc) caused by ⍺-synuclein aggregation. The use of secretomes released by medicinal signaling cells (MSCs) is one the promising preventive approaches that target several mechanisms in the neuropathology of PD. Its components target the lack of neurotrophin factors, proteasome dysfunction, oxidative stress, mitochondrial dysfunction, and at last neuroinflammation via several pathways. The complex and obscure pathology of PD induce the difficulty of the search of potential preventive approach for this disease. We described the potential of secretome of MSC as the novel preventive approach for PD, especially by targeting the said major pathogenesis of PD.

## Highlights

• Secretome targets the major pathogenesis of PD.
• Secretome regulates inflammation by balancing pro- and anti-inflammatory cytokines.
• Secretome induces autophagy providing cytoprotective effects.
• Secretome has anti-oxidative, neuroprotective, and neurotrophic due to neurotrophic factors as its component.

## 1 Introduction

Parkinson’s disease (PD) is the second most common progressive neurodegenerative disease, characterized by tremors, rigidity, akinesia/bradykinesia, and postural instability. Patients with PD have a family history of disease in 15%, with 5–10% having monogenic Mendelian inheritance [1]. Neuropathology of PD is characterized by the apoptosis of neurons in the substantia nigra pars compacta (SNpc) and other areas via several pathways caused by ⍺-synuclein aggregation in the form of Lewy body cytoplasmic inclusions, contributing to the symptoms of Parkinson’s Disease [2]. PD is asymptomatic in the early neuronal death of the SNpc, mainly due to compensation of the striatum [3].

Although several mechanisms have been described, the complex pathogenesis of PD remains to be elucidated [[2], [3], [4]]. Some primary mechanisms involving the apoptosis of the dopaminergic (DA) neurons in PD are: the lack of neurotrophic factor [5], proteasome dysfunction [4] oxidative stress [6], mitochondrial dysfunctions [7], and neuroinflammation [8]. The pathology of PD is attributable to the combined influence of hereditary and environmental factors [3]. Therapy to target neuropathology in PD remains to be explored. One of the promising preventive advances is the use of secretomes released by medicinal signaling cells (MSCs) to target several mechanisms in the neuropathology of PD.

The secretome is a secretion of bioactive molecules produced by MSCs that have several effects on cells and tissues [9,10]. Soluble factors of MSCs can modulate most of the neuropathology in PD, such as the immune system, inflammation, apoptosis, and anti-oxidant activities; moreover, it can also induce cell proliferation [10]. Therefore, the neuropathology of PD, such as neurotrophic factor, proteasome dysfunction, oxidative stress, and neuroinflammation, will be discussed further in this review, along with MSC secretome as a potential preventive approach to the debilitating disease.

## 2 Neuropathology of Parkinson’s disease

### 2.1 Neurotrophic factor

Neurotrophins (also known as neurotrophic factors) are proteins secreted by neurons and neuron-supporting cells that regulate the growth and differentiation of the nervous system [5]. The presence or absence of neurotrophic factors determines whether neurons survive or die. [5] Several neurotrophins have been discovered, such as nerve growth factor (NGF), brain-derived neurotrophic factor (BDNF), neurotrophin-3 (NT-3), neurotrophin-4 (NT-4), and others [5]. All neurotrophins have binding specificity to the tyrosine receptor kinase (Trk) family and pan-75 neurotrophin receptors (p75^NTR^), and all have specificities to different Trk receptor subtypes [5]. For example, NGF binds to TrkA, BDNF, and NT-4 binds to TrkB, while NT-3 binds to TrkC [5].

After neurotrophins bind to their Trk receptors, they activate and trigger the Ras/ERK and PI3K pro-survival pathway [5]. The p75NTR binds to all neurotrophins equally and can increase the affinity of TrkA for NGF, increase intracellular ceramide levels, and activate the NFκβ pro-survival gene and JNK kinase pro-apoptotic gene transcription [5]. The p75^NTR^ can bind to pro-neurotrophins and lead to apoptosis [5].

NGF is important in the survival and function of cholinergic neurons of the basal forebrain complex [11]. NGF is derived from proNGF, the product of *Ngf* gene expression [11]. NGF and its receptors are present in melanin-positive neurons of the SNpc. Thus the activation of proNGF-p75^NTR^ activates the pro-apoptosis pathway, and NGF-TrkA activates the pro-survival pathway [12]. Cellular survival and apoptosis depend on relative amounts of proNGF and NGF in nerve tissues and receptors [12].

BDNF can modulate synaptic plasticity and promote neuronal survival when it binds to the TrkB receptor, but it causes apoptosis of DA neurons when downregulated [13]. BDNF is a potential biomarker to support diagnosis and monitor therapy efficacy in brain disorders [14]. Overexpression of BDNF leads to neurogenesis [13], and it is widely expressed in central motor structures, such as basal ganglia, cerebellum, and brainstem [15]. Reduced BDNF, on the other hand, compromises the survival of neurons and the environment, causing susceptibility to the insult and other harmful effects on the nigrostriatal DA nervous system [16]. BDNF/TrkB signaling can inhibit nigrostriatal apoptosis [15], primarily via pro-survival Ras/ERK and PI3K/Akt pathways [5]. Peripheral BDNF levels are reduced in PD patients, and levodopa therapy can stimulate BDNF levels [17]. BDNF level in peripheral blood lymphocytes was associated with disease duration, the severity of impairment, and levodopa therapy [18]. Circulating BDNF is thought to be of central nervous system (CNS) origin to proxy its expression in the CNS [19].

In patients with advanced PD, the level of BDNF is paradoxical, and its cause is still unclear [18]. Two emerging theories explain the paradoxes: dopamine replacement therapy and compensatory mechanism theories. In dopamine replacement therapy theory, prolonged use effect of levodopa (dopamine agonist) medication can stimulate BDNF secretion, causing an increase of BDNF in advanced PD as advanced PD patients are primarily on levodopa medication. In dopamine replacement therapy theory, prolonged use effect of levodopa (dopamine agonist) medication can stimulate BDNF secretion, resulting in an increase in BDNF in advanced PD patients who are primarily on levodopa medication [19]. In advanced PD, a compensatory mechanism in response to the progressive loss of DA neurons in the SNpc is reflected in the greater BDNF later [20].

α-synuclein aggregates can block the BDNF activity by downregulating expression and causing competitive inhibition at the receptor level [19]. It can interact with the TrkB receptor, thus inhibiting the internalization and the distribution of TrkB, causing the signaling from BDNF to be blocked and promoting apoptosis [21]. The α-synuclein causes endoplasmic reticulum stress, increases unfolded protein response, and increases more neurotoxins inside the cell, thereby inducing apoptosis via activation of glycogen synthase kinase 3β (GSK3β), suppression of cyclin D1, and inactivation of AKT [21].

Changes in synthesis transport or signaling can occur as a result of local injury, aging, mutation, or polymorphisms and are correlated with neurodegeneration [5]. Increased neurotrophin expression induces neuroprotection against oxidative stress, excitotoxicity, and apoptosis, while decreased DA areas could induce Parkinson's Disease (PD) [15]. While patients with PD have decreased pro-neurotrophic factors, PD severity correlates positively with pro-neurotrophic factors [22].

### 2.2 Proteasome dysfunction

The ubiquitin-proteasome system (UPS) and the autophagy-lysosome pathway (ALP) are two significant cellular quality control mechanisms of proteasomal function associated with PD. In the UPS, short-lived soluble proteins are tagged by ubiquitin and then degraded by proteasomes, and in the ALP, long-lived macromolecules, cytosolic components, and dysfunctional organelles are degraded. Thus its failure causes the accumulation of aggregated α-synucleins that interfere with proper cellular function leading to PD pathogenesis [4]. Another minor system that affects α-synuclein turnover and metabolism is chaperone-mediated autophagy (CMA).

A study by McKinnon et al. found functional impairment of the UPS in early-onset PD. It preceded neuron losses and motor dysfunction of PD pathogenesis [23]. Proteasome impairment interferes with α-synuclein degradation, causing accumulation and aggregation of α-synuclein [24]. While the overexpression of α-synuclein itself has been associated with inhibition of proteasome activity in several models [23]. α-synuclein inhibits UPS functionality depending on the species of α-synuclein and the cellular environment by specific inhibition through direct binding to 19S and 20 S proteasomes [25] of chymotrypsin-like proteasome function; however, it is not a general mechanism [26]. Proteasome activity is impaired gradually due to aging, leading to aberrant protein aggregates [27]. The nexus of proteasome impairment and α-synuclein accumulation contributes further to PD pathogenesis. Maintaining proteasome function is essential for the degradation of proteins and slowing down neurodegeneration [27].

### 2.3 Oxidative stress

Oxidative stress happens when reactive oxygen species (ROS) production exceeds the clearance by anti-oxidant enzymes and cytosolic chaperones. Accumulation of ROS causes oxidative damage to lipids, proteins, DNA, and RNA depending on the location of ROS production, compromising neuronal function and structural integrity [6]. The brain is rich in fatty acids, and thus is prone to peroxidation, leading to increased radical formation [28]. Higher iron levels in the central nervous system (CNS) are attributable to elevated mitochondrial enzymes, which produce more reactive oxygen species (ROS), increasing susceptibility to oxidative stress [29]. Uncontrolled ROS generation is one of the causative factors in neuronal apoptosis, further causing progressive neurodegeneration [6].

High oxidative stress causes vulnerability to oxidative insult in SNpc DA neurons, further deteriorating oxidative balance [6]. Intracellular RONS levels are regulated by SOD and glutathione (GSH) [30]. In patients with PD, disruption of RONS balance causes oxidative stress, thus damaging the mitochondria and causing mitochondrial dysfunction, and excessive ROS causes lipid peroxidation, DNA damage, and protein oxidation [28]. Increased oxidative stress response and concentration are potential targets for therapeutic strategies [31].

Mitochondrial complex inhibition can cause an increase in ROS production, potentially leading to a DA loss in PD [29]. Rotenone, a selective SN complex I mitochondria inhibitor, supports the involvement of complex mitochondrial inhibition in PD pathogenesis [29]. Inhibition of the mitochondrial complex increases intracellular ROS generation, leading to further damage in mitochondria and, with more substantial inhibition, magnifies ROS production in a vicious cycle [29].

Mitophagy and biogenesis balance the healthy mitochondrial pool and bioenergetic functions to promote homeostasis; consequently, disruption can contribute to mitochondrial dysfunction and PD [32]. A post-mortem investigation revealed mitochondrial complex I and complex II impairment in the substantia nigra neurons of patients with PD [7]. This respiratory chain complex dysfunction is related to the increased generation of ROS when electrons prematurely escape from the electron transport chain [33].

The mutation in mitochondrial DNA (mtDNA) also has a role in the pathogenesis of PD as multiple mtDNA deletions were seen in SNpc DA neurons of patients with PD, especially in mitochondrial transcription factor A (TFAM) [28]. Mitochondrial DNA is prone to ROS attack due to its proximity to ETC complexes and its lack of histone protein protection [29].

Mitochondria are essential organelles for Ca^2+^ storage and regulation of cellular function [34]. Maintenance of the Ca^2+^ gradient depends on the transmembrane potential integrity across the inner mitochondrial membrane; thus, increases in mitochondrial Ca^2+^ are associated with an accumulation of ROS, especially in DA neurons with frequent exposure to calcium influx [29]. ETC damage and oxidative stress are exacerbated by the destructive loop of Ca2+ excess and ROS generation.

Facilitation of mitochondrial permeability transition pore (mPTP) increases the permeability of Bcl-2-associated X protein (Bax) and Bcl-2 homologous killer (Bak) into mitochondria and disrupting transmembrane potential, thus inhibiting ATP production and further increasing ROS [29]. mPTP opening also facilitates the release of mitochondrial apoptogenic proteins, such as cytochrome *c*, into the cytosol [29]. α-synuclein accumulation in DA neuron mitochondria reduces complex I activity and increases ROS generation before dopamine loss [35].

### 2.4 Neuroinflammation

Neuroinflammation has a significant role in neurodegenerative disease pathogenesis, including PD. In a neuroinflammatory state, glial cells release pro-inflammatory and neurotoxic substances, which contribute to neuronal damage and, eventually, neurodegeneration [36]. Activation of microglia changes its morphology from ramified microglia to amoeboid microglia, which can scavenge like macrophages. M1-type microglia are stimulated by interferon (IFN)-γ and lipopolysaccharide (LPS) in pro-inflammatory states. In contrast, M2-type microglia contributes to cell and tissue repair, regeneration, and remodeling, as also the regulation of immune responses [37]. Microglial activation via IL1B and *Mif* is regulated by microglial autophagy via the PDE10A pathway [38]. Autophagy inhibition enhances PDE10A levels, eliminating the inhibition of NLRP3 inflammasomes due to the inhibition of cyclic AMP (cAMP), and causes microglia to release IL1B and MIF neuroinflammatory cytokines [38]. The neuroinflammatory state also activates astrocytes A1 and induces pro-inflammatory cytokines via M1-microglia, damaging DA neurons and instigating neurodegeneration. Conversely, A2 astrocytes protect neurons by upregulating neurotrophic factors [37].

The activation of microglia by LPS causes selective degeneration of DA neurons, sparing the γ-aminobutyric-acid-ergic (GABAergic) and serotonergic neurons [39]. Infiltration of T lymphocytes and activation of microglia causes increased production of pro-inflammatory cytokines and chemokines, resulting in degeneration of DA neurons due to accumulation of α-synuclein [8]. Although evidence from several studies suggests a destructive effect of the immune response mechanism in PD, its translation to therapeutic interventions to halt disease progression needs further research and clinical trials [40].

## 3 Medicinal signaling cells (MSCs) and its secretome

### 3.1 Medicinal signaling cells

MSCs can originate from some tissues, such as bone marrow, adipose tissue, birth-derived tissues such as umbilical cord and Wharton’s jelly, amniotic fluid and placenta, dental pulp, peripheral blood, synovium, endometrium, skin, and muscle [41]. MSCs (both autologous or allogeneic) can be further divided into two types based on their source: adult MSCs (bone marrow, adipose tissue, peripheral blood, dental pulp), and neonatal tissue-derived MSCs (placenta, amnion, and umbilical cord) [42]. Hypothetically, MSCs may be acquired from virtually any tissue in the human body; yet, limitations exist due to the procurement method's complexity and invasiveness, as well as various donor factors. The vast possibilities of cell therapy have raised expectations for this therapeutic modality. However, the current understanding of its mechanisms and studies on its application is still under investigation. MSCs accomplish their desired effects through multipotentiality, immunomodulatory, pro-regenerative signaling, anti-inflammatory, anti-apoptosis, and pro-angiogenesis processes [43]. MSCs can provide a beneficial milieu and protect and restore injured DA neurons [44]. It has been found that local or systemic MSC delivery with autologous or allogeneic bone marrow MSCs can improve cardiac function and functional capacity, thus improving the patient’s clinical condition and quality of life [44]. MSCs also play a role in cell replenishment, anti-inflammation, and immunomodulation in patients with PD and other neurodegenerative diseases [45]. More specifically, bone marrow MSCs secrete cytokines and trophic factors to protect and stimulate the regeneration of DA neurons and the differentiation and migration of resident cells in the substantia nigra and subventricular zone. The study by Abdelwahab et al. [46] found that the MSC secretome is superior in neuroprotection and prevention of α-synuclein aggregation neuroprotection.

However, the multipotentiality of MSCs can be a double-edged sword as in the case of colorectal carcinoma; it secretes cytokines (IL-6 and Angiopoietin-1) which induces cancer cells to secrete endothelin-1, activating AKT and ERK pathways, leading to mobilization and tumor formation. Furthermore, MSCs can support vasculogenic mimicry in patients with melanoma, thereby reinforcing tumor vasculature. In hepatocellular carcinoma, MSCs can promote the epithelial–mesenchymal transition by promoting the metastasis and dissemination of cancer [47].

### 3.2 Secretome

MSCs’ secretome supports the trafficking, adhesion, and endocrine signaling of molecules. Composed of a lipid bilayer with proteins enveloping bioactive molecules such as enzymes, genetic materials (DNA, RNA, and microRNA), signal transduction proteins, immunomodulatory signaling, and growth factors, they are divided by diameter into apoptotic bodies (>1000 nm), microvesicles (100–1000 nm), and exosomes or multivesicular bodies (30–200 nm) [48]. Among the secretome, exosomes have gained the most attention. They are akin to a complicated cargo of bioactive chemicals, including RNAs (miRNA, rRNA, and lncRNA), lipids like prostaglandins, and protein enzymes. On another note, exosomes can rapidly (within 30 min) be distributed within the spleen, liver, and lungs and be excreted by renal and hepatic processes in one to 6 h [48].

## 4 Discussion: medicinal signaling cell (MSC) secretome as a potential preventive approach

**Fig. 1:**
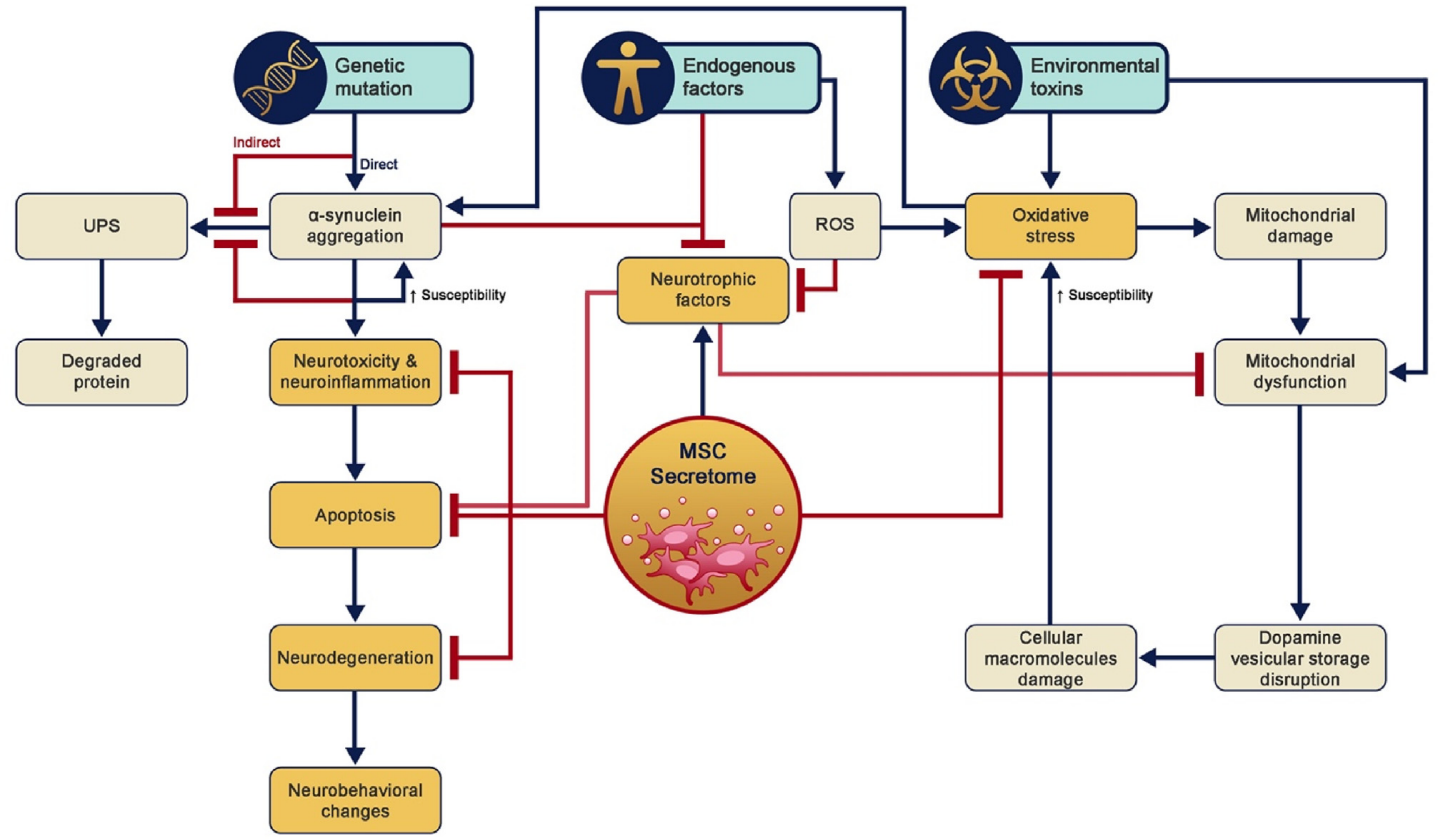
Secretome as neuropathology-targeted intervention of Parkinson’s disease.

Secretome is the secretion of bioactive molecules and extracellular vesicles (ECV) by MSCs, such as cytokines, chemokines, growth factors, and anti-inflammatory factors, that produce numerous paracrine effects on cells and tissues [9,10]. Soluble factors of MSCs can modulate the immune system, inflammation, and autophagy [10]. The factors also have anti-apoptotic, pro-angiogenic, anti-microbial, and anti-oxidant activities, while also inducing cell proliferation [10]. Despite the complex mechanisms underlying PD neuropathology, MSC secretome has the potential to target neuropathology in patients with PD via many pathways as a cell-free therapy (Fig. 1). The advantage of utilizing secretome over stem-cell-based therapies are; immune compatibility, reduced tumorigenicity, decreased risk of emboli formation, and minimized transmission of infections [49].

Secretome anti-inflammatory effects are mediated by soluble immunoregulatory molecules, such as interleukin (IL)-13 and IL1 receptor antagonist (IL1RA), but they also contain pro-inflammatory cytokines such as IL1β [49]. The balance between these cytokines determines the final effect of the secretome. The human umbilical cord embryonic stem cell (hUCESC) secretome reduces the expression of pro-inflammatory cytokines (IL-6, IL-8, and TNFα) while increasing the expression of anti-inflammatory cytokines (IL-10) [50]. Secretome from human umbilical cord MSC has a role in autophagy regulation. A study by Chen et al. found that the secretome induces autophagy, thus providing cytoprotective effects after PD induction by 6-OHDA [43]. hUCESC secretome also has an anti-apoptotic effect in normal cells by producing inhibitor proteins of apoptosis (Bcl-2) and reducing the expression of pro-apoptotic proteins (Bax) [49].

Neurotrophic factors present in the MSC secretome generate antioxidative [51], neuroprotective, and neurotrophic effects. A previous study found that the human bone marrow MSC (hBMSC) secretome normalizes the defective process (proteostasis and altered gene transcription) in PD, resulting in neuroprotective effects in an animal model due to secretion of molecules related to UPS and histone systems [53].

Mendes-Pinheiro et al. [53] found that the 6-OHDA rat model treated with hBMSC secretome showed a better success rate of eaten pellets in the staircase test, which assessed forelimb and skilled motor function compared to those treated with the staircase test in the untreated group, but the same was not seen in the animals receiving hBMSC transplant. The hBMSC secretome was further characterized using a non-biased proteomic analysis using combined mass spectrometry (MS), and it was discovered that Parkinson's disease-related proteins were among the most abundant in terms of signaling pathways, suggesting a beneficial effect for the PD model with elements of the proteasome as one of the most represented protein complexes. This study showed the disease-modifying effect of secretome by modulating the pathophysiology of PD.

Another study by Teixeira et al. [51] found that the transplantation of the hMSCs secretome resulted in the survival of DA neurons in the 6-OHDA PD model. The secretome in this study was further characterized, and it was found to contain essential neurotrophic factors (e.g., BDNF, IL-6) and Cystatin C and galectin-1, which is essential for migration, differentiation, neuroprotection, and anti-apoptotic agents [54]. Mendes-Pinhiero et al. [55] also found similar results by using human neural progenitor cells (hNPCs) secretome transplantation of 6-OHDA rat model that presented better motor performance with higher TH expression level in the secretome of hNPCs compared with the hNPCs itself. The secretome of this study was characterized further and found that it consisted of galectin-1, and cystatin C, which was linked to the NF–κB signaling cascade, which induces expression of BDNF, a protein crucial for neuronal protection and survival. A similar result was also demonstrated in the study by Chen et al., which found that the secretome of stem cells from human exfoliated deciduous teeth (SHED) can significantly improve the phenotype of PD and also improves motor deficiency, and also restore DA neuron loss by rotenone via anti-inflammatory effects and upregulation of neurodevelopment and nerve regeneration [56].

Despite the beneficial effects of the secretome in targeting the neuropathology of PD, most of the mechanisms by which secretome modulates disease progression have not been described in detail. The exact optimal dose of secretome required has yet to be determined. More research is needed to establish the precise mechanism by which these molecules affect and target the neuropathogenesis of PD, as well as the appropriate dose. Secretome dosage and mechanism of action should be the focus of future research.

## 5 Conclusion

The obscure and complex pathogenesis of PD has yet to be unraveled, but the need for a novel preventive approach must be addressed promptly. Some of the most crucial neuropathology of PD are related to neurotrophins, proteasome dysfunctions, oxidative stress, and ultimately neuroinflammation. MSC secretome is one of the most promising preventive approaches as it contains numerous bioactive components that target prominent neuropathologies of PD. More research is needed to further elaborate on the effect of MSC secretome as a cell-free therapy and disease-modifying agent for PD.

## Declaration of competing interest

The research was funded by Jakarta in Focus research (2020) provided by Research and Community Service Center, Atma Jaya Catholic University of Indonesia.
